# Dr. John Huntington Van Kleeck

**Published:** 1916-07

**Authors:** 


**Dr. John Huntington Van Kleeck,** Buffalo 1873, died at his home in Brooklyn, of pneumonia, May 7.

